# The natural compound n-butylidenephthalide kills high-grade serous ovarian cancer stem cells by activating intrinsic apoptosis signaling pathways

**DOI:** 10.7150/jca.51650

**Published:** 2021-03-30

**Authors:** Yu-Hsun Chang, Kun-Chi Wu, Dah-Ching Ding

**Affiliations:** 1 Department of Pediatrics, Hualien Tzu Chi Hospital, Buddhist Tzu Chi Foundation, and Tzu Chi University, Hualien, Taiwan.; 2 Department of Orthopedics, Hualien Tzu Chi Hospital, Buddhist Tzu Chi Foundation, and Tzu Chi University, Hualien, Taiwan.; 3 Department of Obstetrics and Gynecology, Hualien Tzu Chi Hospital, Buddhist Tzu Chi Foundation, and Tzu Chi University, Hualien, Taiwan.; 4 Institute of Medical Sciences, Tzu Chi University, Hualien, Taiwan.

**Keywords:** Butylidenephthalide, high grade serous ovarian cancer, cancer stem cell, ALDH, apoptosis.

## Abstract

High-grade serous ovarian cancer (HGSOC) constitutes 80% of ovarian cancer. Cancer stem cells (CSCs) are responsible for most of the tumor metastasis and chemoresistance. n-Butylidenephthalide (BP) is a potential anti-tumor agent for treating a variety of cancers. The aim of this study was to evaluate the effect of BP on CSCs of HGSOC. CSCs were isolated using the CSC marker (ALDH; aldehyde dehydrogenase) from KURAMOCHI and OVSAHO cells (HGSOC cell lines). The cell proliferation, IC50 (the half-maximal inhibitory concentration), cell migration and invasion, TUNEL (terminal deoxynucleotidyl transferase (TdT) dUTP nick end labeling) assay, western blot of ovarian CSC were evaluated. The animal xenograft studies were evaluated on an immunodeficient mouse model. The results showed the proliferation of ALDH+ cells was greater than that of ALDH- cells. The dosage of IC50 of BP was higher in ALDH+ cells than in mixed cancer cells (317.2 vs. 206.5 μg/ml) in KURAMOCHI cells, but not in OVSAHO cells (61.1 vs. 48.5 μg/ml). BP could inhibit the migration and invasion of both cancer stem cells. BP treatment could activate apoptosis signaling, as indicated by the TUNEL assay and the increased expression of cleaved caspase-3, -7, and -9 but not cleaved caspase-8. A low dose of BP (20 and 25 μg/mL) treatment could increase the toxicity of taxol and cisplatin. In the animal model, BP (200 mg/kg) treatment also decreased the KURAMOCHI and OVSAHO tumor growth rate and induced tumor apoptosis. In conclusion, BP could kill ALDH+ CSCs of HGSOC *in vitro* and *in vivo* by inducing apoptosis. BP may provide a new therapeutic approach for HGSOC.

## Introduction

Ovarian cancer is the deadliest cancer in women. Although its incidence is not high, ranking 7th in Taiwanese women, more than 1400 new cases are diagnosed each year 1. Recently, the survival rates of patients with ovarian cancer have moderately improved with advances in drug therapies 2. However, despite the development of screening programs in the United Kingdom and the United States, mortality associated with ovarian cancer has not improved 3,4.

Tumors are composed of cells with varying degrees of malignancy. Tumor development is mediated by specialized, pluripotent, and self-proliferating cells that have tumorigenic properties and are known as cancer stem cells (CSCs) 5. They are often resistant to traditional treatment 6. Thus, drugs that specifically target CSCs are of particular interest. The CSC of HGSOC 5,7, the most prevalent form of ovarian cancer 8, can be identified and isolated for study using aldehyde dehydrogenase activity (ALDH) as a marker 5.

The current treatment for ovarian cancer involves debulking surgery and adjuvant chemotherapy with platinum and paclitaxel 9. Irrespective of this treatment, the recurrence rate of ovarian cancer is high. Resistance to the current chemotherapeutic drugs may develop after multiline chemotherapies 10. In addition, the toxicity of these drugs is high, precluding some patients from completing therapy. Therefore, the development of new drugs or drugs that increase the efficacy of currently available drugs is warranted.

*Angelica sinensis* is a common Chinese herbal medicine used for the treatment of cough, headache, and angina and to strengthen muscles 11. The active ingredient in the chlorinated layer of *A. sinensis* was identified to be n-butylidenephthalide (BP). BP has been used to treat a variety of cancers, including the brain 12, lung 13, liver 14, and gastric 15. BP inhibits telomerase activity and affects the proliferation of malignant brain tumor cells. BP was also found to stimulate expression of the receptor tyrosine kinase AXL 16, Nur77 in the orphan nuclear receptor 17, and S-phase kinase-associated protein 2 (Skp2) 18.

However, the effect of BP on HGSOC has not yet been determined. This study aims to determine whether BP can kill HGSOC CSCs and whether BP co-treatment with common chemotherapeutic agents increases their effectiveness.

## Methods

### Cell culture

The HGSOC cell lines (KURAMOCHI and OVSAHO cells) were used in this study and purchased from Japan Cell Bank. Both cell lines were confirmed gene expressions mimicking HGSOC 19. The cell line was maintained in DMEM (Sigma, St. Louis, MO, USA) supplemented with 10% FBS, 0.1% non-essential amino acids (NEAA), 2 mM L-glutamine, and 1% penicillin-streptomycin. The cells were incubated at 37 °C with 5% CO_2_.

### Isolation of ovarian CSC by flow cytometry

We used cell fluorescence-activated cell sorting (FACS) to isolate ALDH+ cells from the KURAMOCHI and OVSAHO cell lines 20. The Aldefluor assay kit (Stem Cell Technologies, Cambridge, MA, USA) was used to determine the ALDH activity. The activated ALDEFLUOR reagent was used to trypsinized and incubated with cells for 50 min at 37^o^C. Cells were incubated with inhibitor DEAB as control cells to identify for ALDH+ and ALDH- cell populations. Then BD FACSVerse flow cytometer (BD Biosciences, San Jose, CA, USA) was used to examine and analyze all the stained cells. The BD FACSAria Fusion flow cytometer (BD Biosciences) was used for sorting ALDH+ cells. After sorting, ALDH+ and ALDH- cells propagated using the above culture medium for no more than five passages. According to our analysis, the percentage of ALDH+ cells was more than 80% within 5 passages.

### Chemicals and antibodies

BP (Sigma-Aldrich) was dissolved in vitamin K solution (Sigma-Aldrich). The stock solution was with a concentration of 200 μg/μl. The following antibodies were used: caspase 3, 7, 8, 9 (Cell Signaling Technology, Danvers, MA, USA); tubulin and beta-actin (Abcam, Cambridge, UK). The secondary antibodies goat anti-rabbit and -mouse were purchased from Cell Signaling Technology. The FITC conjugated secondary antibody was purchased from Sigma.

### Assessment of cell viability

Cell viability was determined by XTT assay (Biological Industries Ltd., Kibbutz Beit Haemek, Israel) used as instructed by the manufacturer. We seeded 2 × 10^3^ cells/cm^2^ in 96-well plates with different concentrations of BP (0, 15, 30, 60, 120, 240 μg/ml for ordinary KURAMOCHI cells, and 0, 12.5, 25, 50, 100, 200, and 400 μg/ml BP for ALDH+ KURAMOCHI cells, 0, 12.5, 25, 50, 100, 200 μg/ml for both OVASAHO ALDH+ and ALDH- cells) for 48h. Then the half-maximal inhibitory concentration (IC50) of both types of cells was obtained. The four-parameter logistic regression (4PL) method described in the previous literature was used 21. The equation is expressed as follows: Y=d+(a-d)/(1+(X/c)^b^), where Y is the response, and X is the concentration. The variable a is the bottom of the curve, and d is the top of the curve. The variable b is the slope factor, and c is the concentration corresponding to the response midway between a and d 22. The XTT solutions and N-methyl dibenzopyrazine methyl sulfate (PMS) were defrosted immediately in a water bath at 37°C. To each 100-μL culture in wells of 96-well plates was added 50 μL XTT/PMS. After 2-5 h of incubation at 37°C, plates were analyzed by spectrophotometry to determine the optical density of the solutions at a wavelength of 450 nm (reference wavelength, 650 nm).

### Assessment of cell migration

Tumor cells (5 × 10^4^ cells) were seeded into the top well of a Boyden chamber (24-well transwell) with a pore size of 8 μm (Costar, Corning Inc., Corning, NY, USA). The ALDH+ cells migrate toward the lower well filled with the same culture medium with or without BP (100 or 200 μg/ml for KURAMOCHI, 25 or 50 μg/ml for OVSAHO). After 48 hours of migration, crystal violet (Sigma) was used to stain the migrated cells. The stained cells were counted using a bright-field microscope. Each experiment was repeated three times.

### Invasion assay

Invasion assays were carried out in Matrigel-coated Boyden chambers (filter pore size, 8 μM) in 24-well plates (BD). In the top wells, the culture medium without serum was seeded with 5 × 10^4^ cells. The bottom wells were added medium with 10% FBS and BP (0, 100, or 200 μg/ml for KURAMOCHI and 25 or 50 μg/ml for OVSAHO). Treatments were added to both upper and lower chambers as indicated. After 24 hours, the free cells were removed gently with a cotton swab. The invading cells were fixed with 4% formaldehyde and crystal violet (Sigma) stained. The slides were air-dried and photographed, and the cells were counted using a bright-field microscope.

### Western blot analysis

Tumor cell and tissue lysates were loaded onto a gradient 5-20% sodium dodecyl sulfate-polyacrylamide gradient gel. After electrophoretic separation, the proteins were transferred to a polyvinylidene difluoride membrane (Bio-Rad). The membrane was blocked at room temperature in a solution of 3% nonfat dry milk in PBS and 0.1% Tween-20 and then rinsed in PBS/0.1% Tween-20. Blots were incubated with diluted solutions of polyclonal anti-caspase 3, 8, or anti-cleaved caspase 3, 7, 8, or 9 antibodies (1:200, St. John's Lab, London, UK) and treated with 1:5000 diluted anti-rabbit immunoglobulin G horseradish peroxidase (HRP) for staining (Amersham GE, Taipei, Taiwan). Beta-actin proteins (1: 200, Santa Cruz Biotechnology, Santa Cruz, CA, USA) were used as internal controls. HRP signals were detected using an electrochemiluminescence kit (Promega, Fitchburg, WI, USA).

### Assessment of the additive chemotherapeutic effect of BP

To determine the killing ability of BP alone or in combination with cisplatin or taxol, we measured the viability of KURAMOCHI and OVSAHO cells (2500 ALDH^+^ cells/well) in 96-well plates. BP (25μg/ml in KURAMOCHI and 20 μg/ml in OVSAHO) with or without cisplatin (5μM or 10 μM) or taxol (10 nM or 50 nM). Each experiment was conducted in triplicate. Plates were incubated at 37˚C with 5% CO_2_ for 72 hours. Viable cells were counted by the XTT assay as described.

### Assessment of BP activity *in vivo*

The animal experiment procedures were approved by the Animal Research and Care Committee of the Buddhist Tzu Chi General Hospital (106-48). All procedures were performed in compliance with the National Institutes Health Guide for the Care and Use of Laboratory Animals. Non-obese, diabetic-severe combined immunodeficiency mice (NOD-SCID) (strain NOD.CB17-Prkdcscid/JTcu) purchased from Tzu Chi University were used for these experiments.

KURAMOCHI and OVSAHO ALDH^+^ cells (1× 10^6^) were injected subcutaneously into the backs of 4-5-week-old female mice. After tumors had grown to a volume of 50 mm^3^, mice were separated into two groups (control and treatment groups; n = 6 per group). Controls were treated with vehicle alone (Vitamine K, 10mg/ml), and the experimental group was treated with different doses of BP (100 or 200 mg/kg) for 5 days. Tumor dimensions (length and width) were measured with calipers and the tumor volume determined using the following formula: volume = 1/2 (length × width^2^).

For histological examination, tumor tissues were fixed in 4% paraformaldehyde. Tumors were cut into 6-μm thick sections and stained with hematoxylin and eosin. Tumor tissues were observed under a microscope at 200× magnification. The morphology and cell density were observed and recorded.

### TUNEL assay

Cell apoptosis was assayed using a terminal deoxynucleotidyl transferase dUTP nick end labeling (TUNEL) Assay Kit (Roche, IN, USA) according to the manufacturer's instructions. For ALDH+ KURAMOCHI and OVSAHO cells, they were seeded with 1 × 10^5^ cells in one well of 12-well culture plates. Cultured cells were allowed to attach for 24 hours. Then we treated BP (200 μg/ml for KURAMOCHI, 50 μg/ml for OVSAHO) for 48 hours. Adherent tumor cells were fixed in 4% paraformaldehyde. For ALDH+ KURAMOCHI and OVSAHO cells xenograft, tumor samples were fixed in formalin and embedded in paraffin, and sectioned with a 3 μm of thickness. TUNEL probes were used to detect breaks in DNA strands, followed by incubation in permeabilization solution for 2 min on ice. Cells were washed twice in PBS and the TUNEL reaction mixture added, followed by incubation at 37°C for 60 min in a humidified atmosphere in the dark. Samples were washed twice in PBS twice and observed under a fluorescence microscope.

### Statistical analysis

Data are presented as the mean ± SD of at least three independent experiments. The Mann-Whitney U test was used to compare two independent variables, and one-way ANOVA with post-hoc analysis with the Bonferroni test was used to compare three independent variables. Statistical analysis was performed using GraphPad Prism 6 (La Jolla, CA, USA). P < 0.05 was considered a significant difference.

## Results

### The characteristics of ALDH+ tumor cells in KURAMOCHI and OVSAHO cells

**Figure 1:**
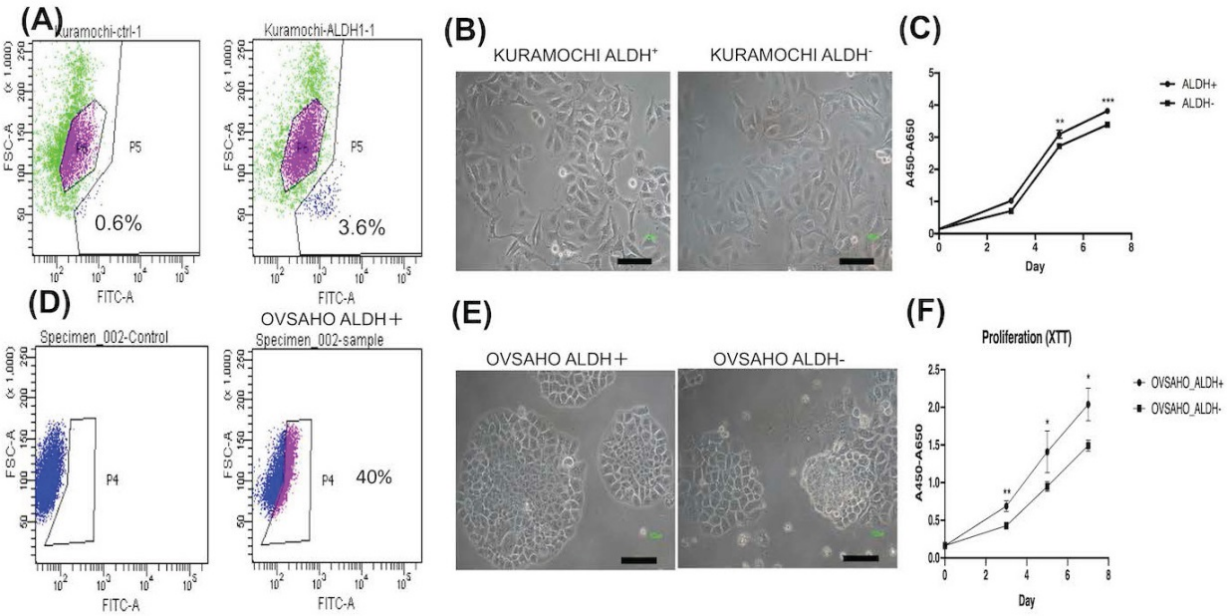
The cancer stem cell (CSC) marker, morphology, and proliferation of KURAMOCHI and OVSAHO cells. (A) ALDH expression, a CSC marker, was identified using the Aldefluor assay and measured by flow cytometry. Data are expressed as dot plots and show the percentage of ALDH^+^ cells in the total population of KURAMOCHI cells. (B) The morphology of ALDH^+^ and ALDH^-^ KURAMOCHI cells. Scale bar = 100 μm. (C) The proliferation of ALDH^+^ and ALDH^-^ KURAMOCHI cells (n=3). (D) The percentage of ALDH^+^ cells in the total population of OVSAHO cells. (E) The morphology of ALDH^+^ and ALDH^-^ OVSAHO cells. Scale bar = 100 μm. (F) The proliferation of ALDH^+^ and ALDH^-^ OVSAHO cells (n=3). *P<0.05, **p < 0.01; ***p < 0.001.

To know the expression of the CSC marker ALDH in both cancers, we used flow cytometry to evaluate. ALDH^+^ cells composed 3.6% in KURAMOCHI cells and 40% in OVASAHO cells (Fig. 1A, D). The morphology of KURAMOCHI ALDH^+^ and ALDH^-^ cells was the same (cobblestone-like appearance) (Fig. 1B). The morphology of ALDH^+^ OVSAHO cells showed more colony formation than ALDH- OVSAHO cells (Fig. 1E). The ALDH^+^ cells proliferate faster than the ALDH^-^ cells in both cancer cells (p < 0.01 in KURAMOCHI, p<0.05 in OVSAHO, Fig. 1C, F). Taken together, these findings show that type 2 ovarian cancer ALDH+ cells owned a faster proliferation rate than ALDH- cells.

### IC50 of BP is higher for ALDH^+^ than ordinary KURAMOCHI cells

**Figure 2:**
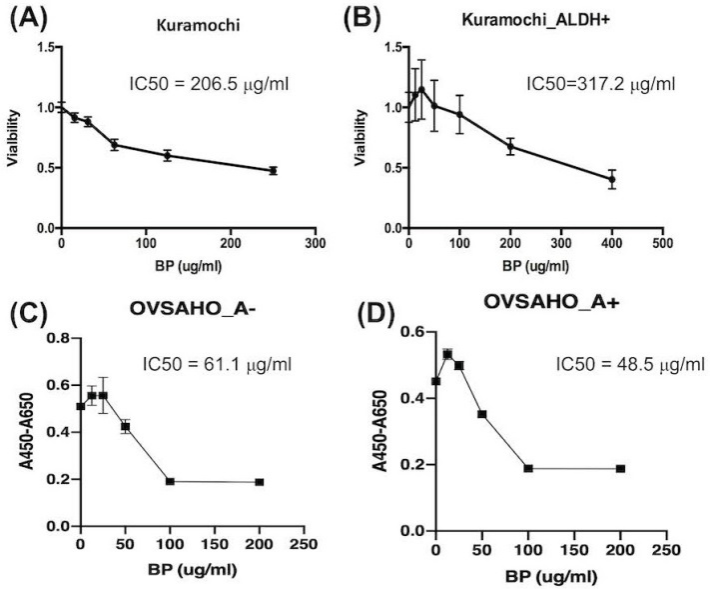
The half-maximal inhibitory concentration (IC50) of BP for ALDH^+^ and ordinary KURAMOCHI and OVSAHO cells after adding different concentrations of BP for 48h. (A) The 2 × 10^3^ cells/cm^2^ tumor cells were plated in one well of a 96-well plate and treated with different concentrations of BP (0, 15, 30, 60, 120, 240 μg/ml). After 48h of culture, the IC50 of BP for the total population of KURAMOCHI cells was calculated as 205.6 μg/ml (n=3). (B) The IC50 of BP for KURAMOCHI ALDH^+^ cells (concentration: 2 × 10^3^ cells/cm^2^) after BP treatment (0, 12.5, 25, 50, 100, 200, and 400 ug/ml) for 48h. The IC50 of BP for ALDH^+^ KURAMOCHI cells was calculated as 317.2 μg/ml (n=3). (C) The 2 × 10^3^ cells/cm^2^ OVSAHO cells were plated in one well of a 96-well plate and treated with different concentrations of BP (0, 12.5, 25, 50, 100, and 200 μg/ml). After 48h of culture, the IC50 of BP for OVSAHO cells was calculated as 61.1 μg/ml (n=3). (B) The IC50 of BP for OVSAHO ALDH^+^ cells (concentration: 2 × 10^3^ cells/cm^2^) after BP treatment (0, 12.5, 25, 50, 100, and 200 μg/ml) for 48h. The IC50 of BP for ALDH^+^ OVSAHO cells was calculated as 48.5 μg/ml (n=3).

The IC50 of BP for ordinary KURAMOCHI cells after 48 h of treatment was 206.5 μg/ml (Fig. 2A), while that for ALDH^+^ cells was 317.2 μg/ml (Fig. 2B). The IC50 for OVSAHO ALDH+ cells after 48 h of treatment was 48.5 μg/ml (Fig. 2C) which was lower than ALDH- cells (61.1 μg/ml, Fig. 2D). This result indicates that a higher IC50 of BP is required to kill KURAMOCHI ALDH+ cells but not OVSAHO ALDH+ cells.

### BP inhibited ALDH^+^ KURAMOCHI and OVSAHO cell migration and invasion

**Figure 3:**
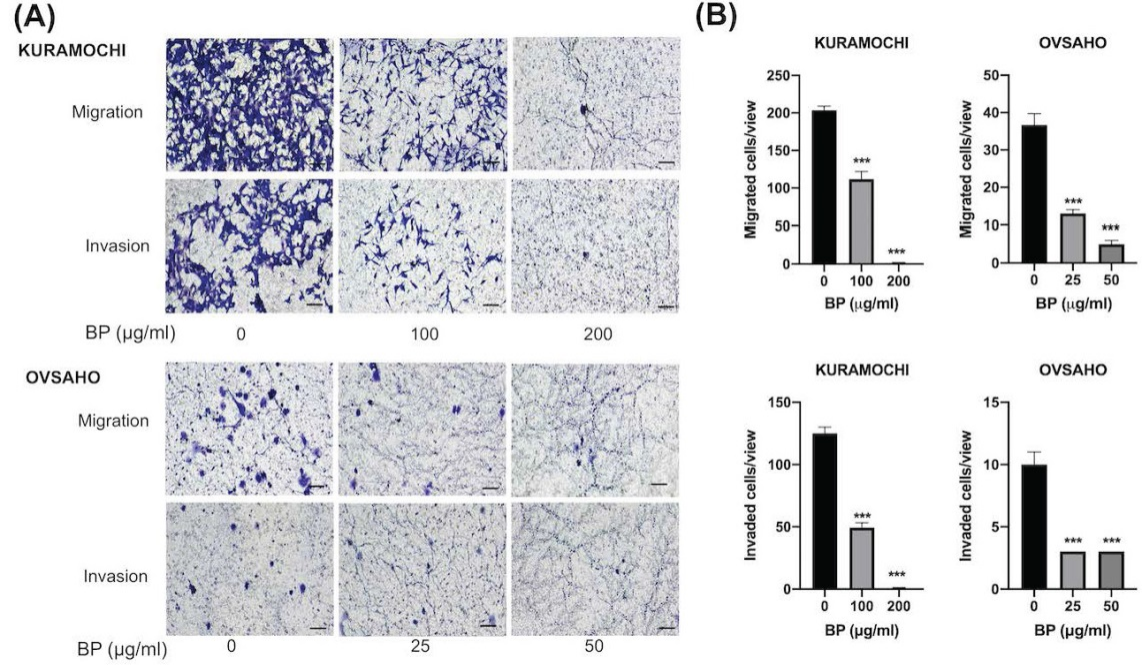
Decreased migration and invasion of ALDH^+^ KURAMOCHI cells and OVSAHO after treatment with BP. (A) Pictures of migration assays of ALDH^+^ KURAMOCHI and OVASAHO cells (5 × 10^4^ cells) with or without the treatment of BP for 48 hours (200 μg/ml and 100 μg/ml for KURAMOCHI [n=3], 25 and 50 μg/ml for OVSAHO [n=3]). Pictures of invasion assay of ALDH^+^ KURAMOCHI and OVSAHO cells (5 × 10^4^ cells) with or without the treatment of BP for 24 hours (200 μg/ml and 100 μg/ml for KURAMOCHI [n=3], 25 and 50 μg/ml for OVSAHO [n=3]). Scale bar = 100 μm. (B) Quantification of migrated and invaded cells per view of KURAMOCHI and OVSAHO after treating them with different concentrations of BP. ***p < 0.001.

To know the BP influence on migration and invasion abilities of ovarian CSC, we did a transwell migration and invasion assay to evaluate. BP treatment significantly decreased KURAMOCHI ALDH+ cell migration at 200 (Fig. 3A) and 100 μg/ml (Fig. 3B) of BP treatment and OVSAHO ALDH+ cell migration at 25 (Fig. 3E) and 50 μg/ml (Fig. 3F) of BP. In the invasion assay, BP treatment significantly decreased the invasion of KURAMOCHI ALDH+ cells at 200 (Fig. 3C) and 100 μg/ml (Fig. 3D) of BP treatment and OVSAHO ALDH+ cells at 25 (Fig. 3G) and 50 μg/ml (Fig. 3H) of BP as compared to untreated cells. These results indicate that BP inhibits the migration and invasion capabilities of ovarian CSCs.

### BP inhibited KURAMOCHI and OVSAHO ALDH^+^ cell proliferation via apoptosis

**Figure 4:**
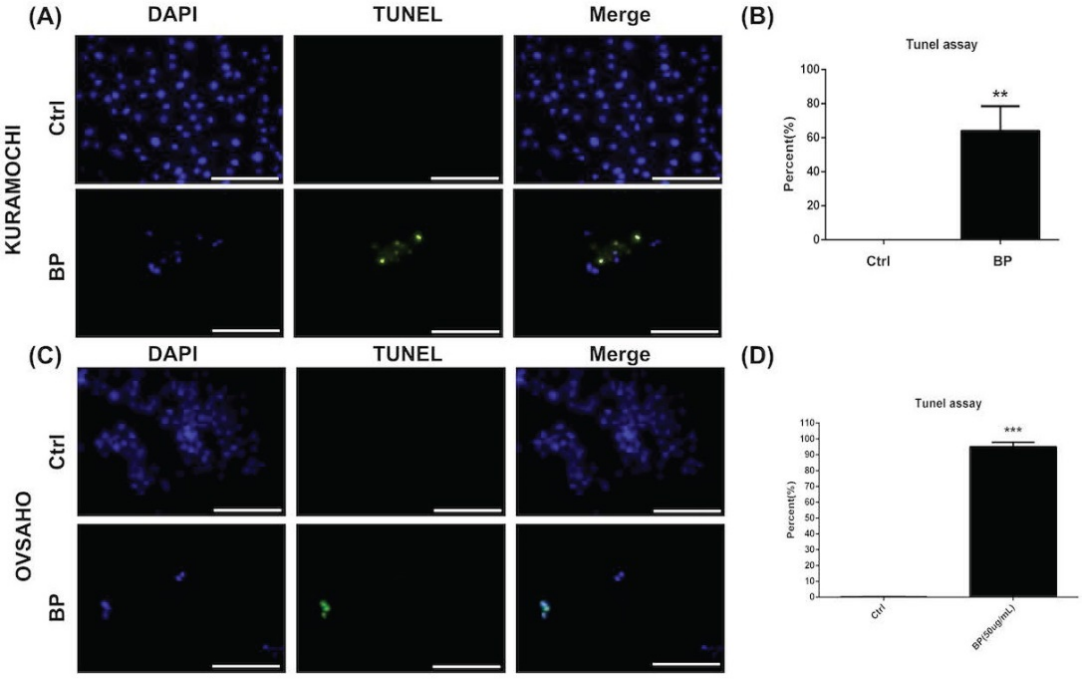
BP inhibited ALDH^+^ KURAMOCHI and OVSAHO cell proliferation via apoptosis. (A) TUNEL assay of ALDH^+^ KURAMOCHI cells with or without BP treatment (200 μg for 48 hours). Scale bar = 100 μm. (B) Quantification of TUNEL^+^ cells in both groups (n=3 each). **p < 0.01. (C) TUNEL assay of OVSAHO cells with or without BP treatment (50 μg for 48 hours). Scale bar = 100 μm. (D) Quantification of TUNEL^+^ cells in both groups (n=3 each). ***p < 0.001.

The level of apoptosis was compared between KURAMOCHI (Fig. 4A, B) and OVSAHO (Fig. 4C, D) ALDH+ cells with and without BP treatment using the TUNEL assay. Many TUNEL^+^ cells were observed after BP treatment (Fig. 4A, C), with significantly more TUNEL^+^ cells with BP treatment than without (p < 0.01 in KURAMOCHI, p<0.001 in OVSAHO, Fig. 4B, D). These results indicate that BP treatment of type 2 ovarian CSC induces cell apoptosis.

### BP treatment activated an apoptosis signaling pathway of KURAMOCHI and OVSAHO

**Figure 5:**
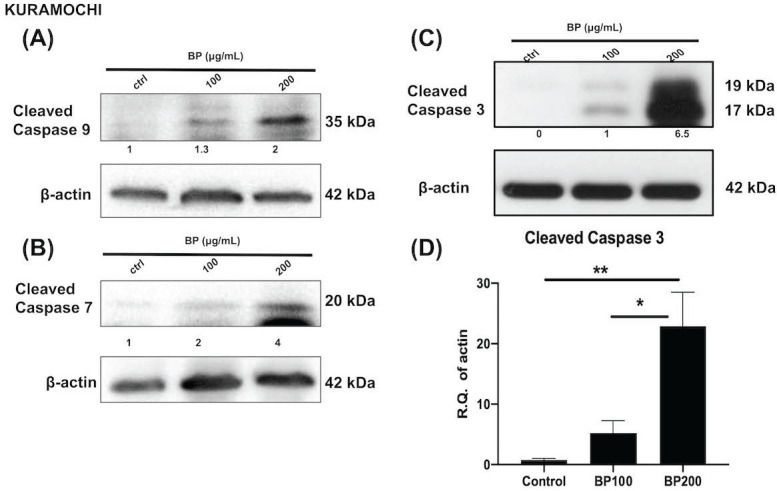
BP treatment activates the apoptosis signaling pathway in KURAMOCHI cells. After BP treatment (100 μg or 200 μg, 48 hours), protein levels of (A) cleaved caspase 9 (B) cleaved caspase 7, and (C) cleaved caspase 3 increased in ALDH^+^ KURAMOCHI cells. (D) Quantification of the protein expression of cleaved caspase 3 (n=4). *p<0.05, **p<0.01. All cropped blots were run under the same experimental conditions. The numbers below each blot revealed the relative quantification to beta-actin.

**Figure 6:**
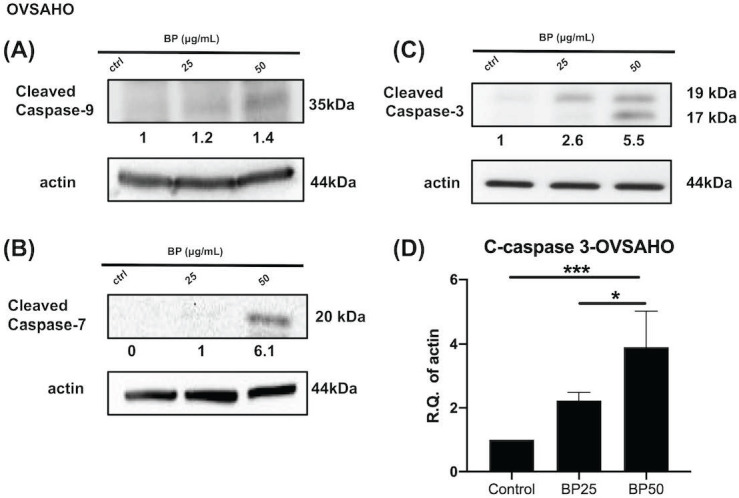
BP treatment activates the apoptosis signaling pathway in OVSAHO cells. After BP treatment (25 μg or 50 μg, 48 hours), protein levels of (A) cleaved caspase 9, (B) cleaved caspase 7, and (C) cleaved caspase 3 increased in ALDH^+^ OVSAHO cells. (D) Quantification of the protein expression of cleaved caspase 3 (n=4). *p<0.05, ***p<0.001. All cropped blots were run under the same experimental conditions. The numbers below each blot revealed the relative quantification to beta-actin.

Next, we investigated the effect of BP on the cell death signaling pathway. BP treatment of KURAMOCHI ALDH^+^ cells (Fig. 5) for 48 hours (100 μg or 200 μg/ml) and OVSAHO ALDH+ cells (Fig. 6) for 48 hours (25 or 50 μg/ml) increased the protein levels of cleaved caspase 9 (Fig. 5A, 6A), caspase 7 (Fig. 5B, 6B), and caspase 3 (p<0.001, Fig. 5C-D, 6C-D). Caspase 8 was not activated in both groups (data are not shown). These results indicate that BP activates the intrinsic apoptosis pathway of type 2 ovarian CSCs.

### BP increases the toxicity of cisplatin and paclitaxel

**Figure 7:**
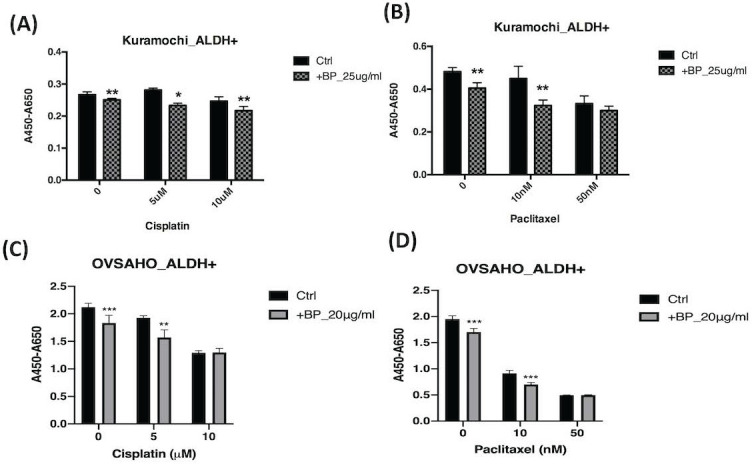
The effects of BP, cisplatin, and paclitaxel treatment on ALDH^+^ KURAMOCHI and OVSAHO cells. (A) ALDH^+^ KURAMOCHI cells treated with BP 25 μg/mL with cisplatin (0, 5, 10 μM). (B) ALDH^+^ KURAMOCHI cells treated with BP (25 μg/mL) with paclitaxel (0, 10, or 50 nM). (C) ALDH^+^ OVSAHO cells treated with BP 20 μg/mL with cisplatin (0, 5, 10 μM). (D) ALDH^+^ OVSAHO cells treated with BP (20 μg/mL) with paclitaxel (0, 10, or 50 nM). All experiments were conducted in triplicate. *p < 0.05, **p < 0.01, ***p<0.001.

To determine whether BP is addicted to the toxicity of other chemotherapeutic agents, we added BP to cisplatin and paclitaxel treatment of type 2 ovarian CSC *in vitro*. Treatment of KURAMOCHI ALDH^+^ cells with BP (25 μg/ml) and OVSAHO ALDH+ cells with BP (20 μg/ml) in combination with cisplatin (5, or 10 μM) resulted in cell viability significantly lower than that of controls (Fig. 7A, C). Next, we investigated the effect of BP (25 μg/ml in KURAMOCHI and 20 μg/ml in OVSAHO) in combination with paclitaxel (0, 10, and 50 nM). With no or low concentration paclitaxel treatment, the number of viable cells was significantly less than that of control cells. However, at a high concentration of paclitaxel, no further decrease in cell viability was observed with BP treatment (Fig. 7B, D). This observation may result from the high toxicity of paclitaxel. Taken together, BP could add to the toxicity of current commonly used chemo drugs.

### BP inhibited xenograft tumor growth via apoptosis

**Figure 8:**
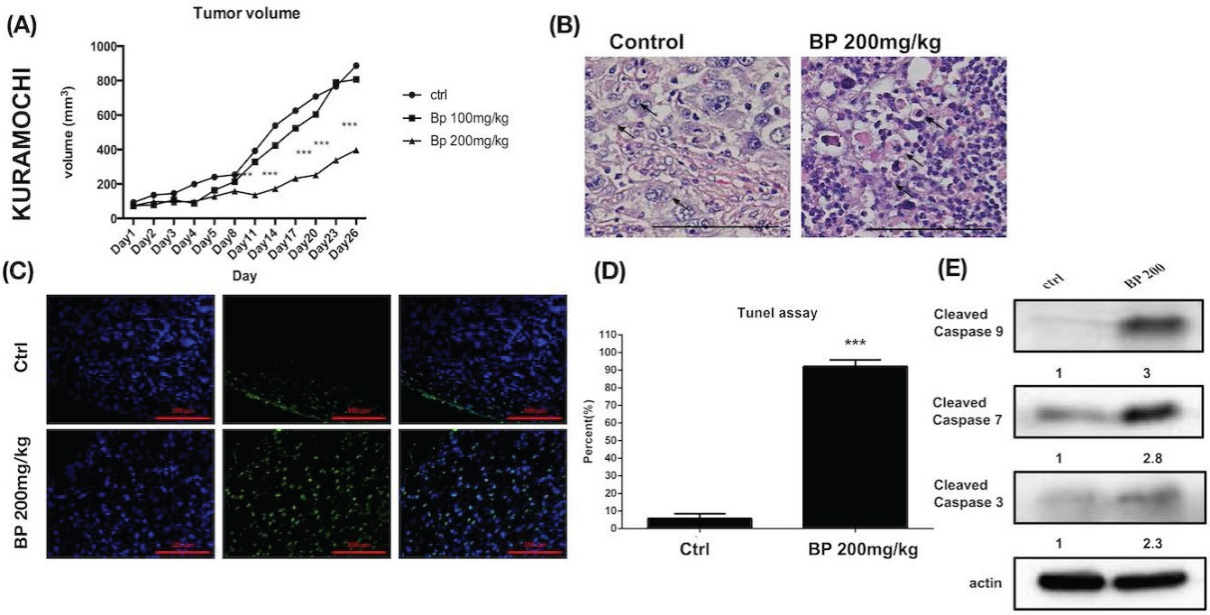
BP inhibited KURAMOCHI cells xenograft tumor growth via apoptosis. (A) KURAMOCHI cells (1 x 10^6^) were injected subcutaneously into the backs of NOD-SCID mice. Tumor growth curves over 26 days are shown with vehicle control and BP treatment (100 or 200 mg/kg for 5 days). The mean relative tumor volumes are shown. ***p < 0.001 (B) Hematoxylin and eosin staining of tumor tissue with or without BP treatment. The Control tumor section showed malignant cells with enlarged round nuclei, coarse chromatin, and prominent nucleoli are seen (arrow). The BP-treated tumor section showed a drop out of cells with inflammatory cell infiltration. The remaining malignant cells show shrunken in size and hyperchromatic pyknotic nuclei (arrow). Scale bar = 100 μm. (C) TUNEL assay of tumor tissue with or without BP treatment. Scale bar = 100 μm. (D) Quantification of TUNEL^+^ cells in both groups (n=3 each). ***p < 0.001. (E) Protein levels of cleaved caspase 3, caspase 9, and caspase 7 were increased in ALDH^+^ KURAMOCHI xenograft. All cropped blots were run under the same experimental conditions. The numbers below each blot revealed the relative quantification of actin.

**Figure 9:**
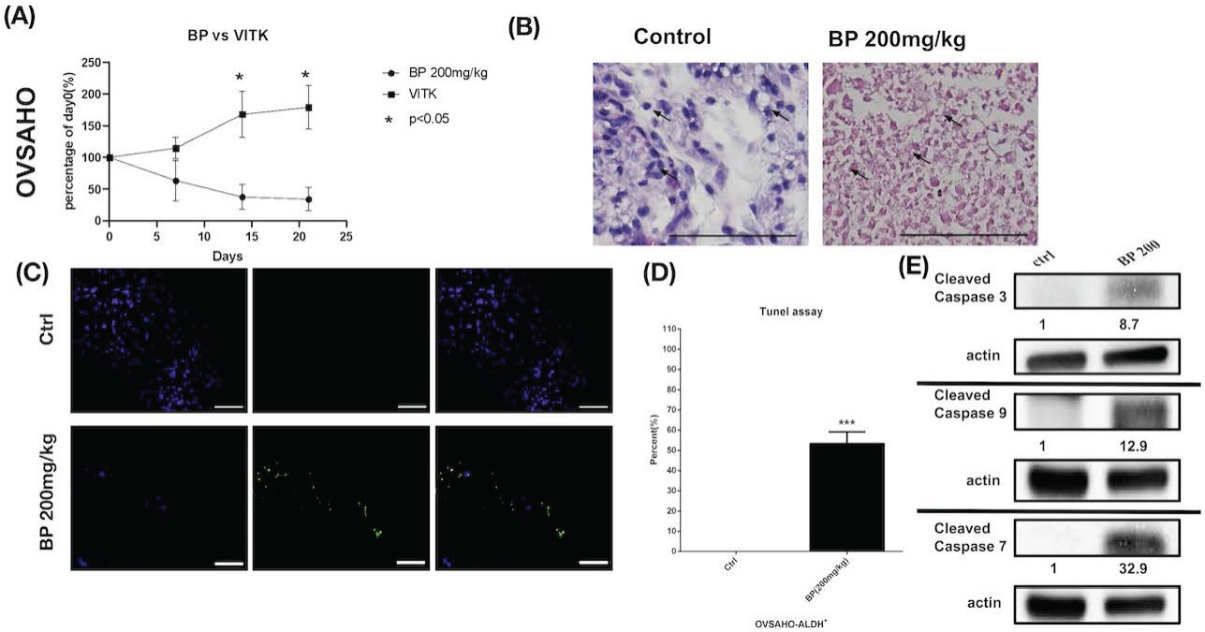
BP inhibited OVSAHO cells xenograft tumor growth via apoptosis. (A) OVSAHO cells (1 x 10^6^) were injected subcutaneously into the backs of NOD-SCID mice (n=6). Tumor growth curves over 21 days are shown with vehicle control (Vitamin E) and BP treatment (200 mg/kg for 5 days). The mean relative tumor volumes are shown. *p < 0.05 (B) Hematoxylin and eosin staining of tumor tissue with or without BP treatment. The control tumor section showed malignant cells with increased nuclear/cytoplasmic ratio (arrow). The BP-treated tumor section showed complete necrosis of cells leaving only an outline of cell shape and cytoplasmic eosinophilia (arrow). Scale bar = 100 μm. (C) TUNEL assay of tumor tissue with or without BP treatment (n=3 each). Scale bar = 100 μm. (D) Quantification of TUNEL^+^ cells in both groups. ***p < 0.001. (E) Protein levels of cleaved caspase 3, caspase 9, and caspase 7 were increased in OVSAHO xenograft. All cropped blots were run under the same experimental conditions. The numbers below each blot revealed the relative quantification of actin.

Human ovarian cancer xenografts were used to investigate the tumor-inhibiting effect of BP in mice. With BP treatment (200 mg/kg) in KURAMOCHI and OVSAHO cells, the tumor volumes were smaller than those of control mice treated with the vehicle (days 11-26 in KURAMOCHI [p < 0.001], day 14 -21 in OVSAHO [P<0.05]) (Fig. 8A, 9A). However, no significant difference was observed between BP (100 mg/kg) and controls in KURAMOCHI cells (Fig. 8A). H & E staining revealed that the nuclei of BP-treated KURAMOCHI xenograft tumors were denser compared with those of the tumors in the control group (Fig. 8B). In the OVSAHO xenograft tumor, there were no nuclei in tumor cells (Fig. 9B). In TUNEL assay of tumor tissue showed significantly more TUNEL^+^ cells after BP (200 mg/kg) treatment (p < 0.001) (Fig. 8C-D, 9C-D). Protein levels of cleaved caspase 3, caspase 9, and caspase 7 were increased in BP-treatment ALDH^+^ KURAMOCHI and OVSAHO xenograft than in control (Fig. 8E, 9E).

Taken together, these results suggest that BP inhibited xenograft growth by activating apoptosis of type 2 ovarian cancer stem cells.

## Discussion

In ovarian cancer, tumor aggressiveness, resistance to therapy, and disease relapse may be determined by a small population of CSCs 23. Therefore, targeting ovarian CSCs is important to cancer treatment 24. Therapies that target both CSCs and cancer cells would have a significant therapeutic advantage 25. An ideal non-toxic, anti-CSC agent might be derived from natural products 26. This study investigates the anti-CSC characteristics of BP, a naturally occurring compound derived from *A. Sinensis*. Our results show that BP significantly inhibited ovarian CSC proliferation *in vitro* and *in vivo*. BP also inhibited ovarian CSC migration and invasion. BP treatment resulted in the death of ovarian CSCs via activation of the apoptosis signaling pathway. BP increased the toxicity of the chemotherapeutic drugs cisplatin and taxol on ovarian CSCs. BP also inhibited the tumorigenicity of CSCs via induced apoptosis in NOD-SCID mice. These findings suggest that BP may be useful for ovarian cancer therapy.

ALDH+ cells are considered to be ovarian CSCs 27-29. Therefore, we isolated ovarian CSC using ALDH as a CSC marker. Different ovarian cancer cells may own a different percentage of CSC (3.6% ALDH+ cells in KURAMOCHI cells and 40% ALDH+ cells in OVSAHO cells). The characteristics of CSC have included self-renewal capacity to enhance tumor initiation, growth, and progression 30. In our study, the proliferation rate was faster in ALDH+ cells than in ALDH- cells noted in KURAMOCHI and OVSAHO cells. The IC50 dosage of KURAMOCHI ALDH+ cells was higher than cancer cells (317 μg/mL vs. 206 μg/mL). A decrease of the tumor-initiating capability of cells isolated from BP-treated tumors indicated anti-CSC effects of BP *in vivo*.

Apoptosis is a targeted cell death program regulated by the caspase protein cascade to prevent inflammatory reactions and damage to surrounding cells 31. The initiator caspases (caspases 8 and 9) activate executioner caspases (caspases 3, 6, 7) to mediate subsequent reactions that activate the expression of key catabolic proteins and enzymes. Apoptosis occurs via extrinsic and intrinsic pathways 31. The extrinsic pathway involves signaling from a ligand to a death receptor, which subsequently activates caspase 8, resulting in the activation of downstream executioner caspases 32. Through cleavage of the pro-apoptotic Bid protein, caspase 8 also activates the intrinsic pathway to induce cell death. Cellular stress activates the intrinsic pathway and then activates mitochondrial cytochrome c to activate caspase 9 33; caspase 9 then induces the expression of executioner caspases. Our study found that BP activates caspases 9, which are associated with the intrinsic pathways. Subsequently, the executioner caspases such as caspase 3 and 7 would be activated to mediate programmed cell death 34. These findings indicate that BP kills CSCs of HGSOC by activating the intrinsic pathway of apoptosis.

Cisplatin and taxol are standard treatments in adjuvant chemotherapy for ovarian cancer 35. Taxol stabilizes microtubules, resulting in mitosis arrest and cell death. Cisplatin is an alkylating agent that kills tumor cells. However, both of these drugs are highly toxic and can cause severe conditions such as urticaria, angioedema, and hypertension. Thus, some patients could not complete the complete cycle of chemotherapy because of severe toxicity 36. Therefore, a research interest focused on lessening the toxicity of chemo drugs was proposed. The previous studies have investigated the use of gold nanoparticles, cucurbitacin B, and proadifen to sensitize ovarian cancer cells to chemotherapeutic drugs 37-39 to allow the use of lower doses of the toxic agents. We found that BP increases the killing effect of cisplatin and taxol on ovarian CSC. This finding demonstrates that BP might be useful for sensitizing target cells to chemotherapeutic drugs, ultimately decreasing their side effects.

This study addresses the possible clinical use of BP for killing ovarian CSCs. However, the concentration of BP required to kill KURAMOCHI cells *in vitro* was higher than we expected. Previous studies showed that the effective BP concentration ranged from 15-67 or 50-84 μg/mL *in vitro* and 300-700 mg/kg *in vivo* 12, 15. In KURAMOCHI cells, we used 100-200 μg/ml *in vitro* and 100-200 mg/ml *in vivo*. We speculated that survival signals may be upregulated during BP treatment in KURAMOCHI cells. This possibility requires further investigation. However, in OVASAHO cells, the IC50 of BP was 48.5 μg/ml in ALDH+ cells which is compatible with previous reports 12, 15. The different cell lines may own different IC50 of BP.

There were two xenograft models that could be used in our study, the subcutaneous xenograft model instead of the orthotopic implantation model. The orthotopic transplantation model could provide a suitable microenvironment and enable tumor cells to develop their malignant behavior 40. The previous study has shown the orthotopic implantation model could recapitulate the clinical scenarios than the subcutaneous xenograft model 40. However, the orthotopic implantation model for ovarian cancer needs surgery to open the abdominal cavity and inject the cell into the ovarian bursa. On the contrary, the subcutaneous xenograft model did not need to open the abdominal cavity and was used widely due to researchers being able to easily detect tumor growth and size. In our study, we used a subcutaneous xenograft model instead of orthotopic implantation of ovarian CSC.

The limitation of this study was to use ALDH as a marker for isolating ovarian CSC. In HGSOC, there are several CSC markers in ovarian cancer such as CD24, CD44, CD117, CD133, and ROR1 41. There is some potential for false positives or negatives. In the future, additional markers could be used for isolating ovarian CSC. In this study, we did not use clinical specimens, which may lack clinical correlation. However, we used an *in vivo* tumor formation experiment to study the *in vivo* effect which might be reflected in the *in vivo* status. Another limitation was the lack of normal cells and other cancer cells as controls. We only used the cells of type 2 ovarian cancer which occupied almost 80% of ovarian cancer. Therefore, the results of these experiments might be applied to 80% of ovarian cancer.

In conclusion, BP kills CSCs of HGSOC via activation of the apoptosis signaling pathway. BP may also be an additive to conventional chemo drugs to lessen the side effect. BP may prove useful in treatments to slow ovarian cancer progression.
